# A new synonym of the Neotropical parasitoid wasp genus *Notiospathius* (Braconidae, Doryctinae), with redescription of two species and description of five new species from Brazil

**DOI:** 10.3897/zookeys.122.1243

**Published:** 2011-08-11

**Authors:** Vladimir Salvador De Jesús-Bonilla, Juliano F. Nunes, Angélica M. Penteado-Dias, Sándor Csösz, Alejandro Zaldívar-Riverón

**Affiliations:** 1 Colección Nacional de Insectos, Instituto de Biología, Universidad Nacional Autónoma de México, 3er. circuito exterior s/n, Cd. Universitaria, Copilco, Coyoacán, A. P. 70-233, C. P. 04510, D. F., México; 2 Programa de Pós-Graduação em Ecologia e Recursos Naturais, Universidade Federal de São Carlos, Rodovia Washington Luís, km 235, 13565-905, São Carlos-SP, Brasil; 3 Universidade Federal de São Carlos, Departamento de Ecologia e Biologia Evolutiva, Rodovia Washington Luís, km 235, 13565-905, São Carlos-SP, Brasil; 4 Hungarian Natural History Museum, Department of Zoology, H-1088 Budapest, Baross u. 13., Hungary

**Keywords:** parasitoid wasps, Brazil, *Notiospathius*, Dorcytinae, Braconidae

## Abstract

A junior synonym of the parasitoid wasp genus *Notiospathius* Matthews and Marsh, *Hansonorum* **syn. n.**, with two new combinations, *Notiospathius carolinae* (Marsh) **comb. n.** and *Notiospathius pauli* (Marsh) **comb. n.**, are proposed. Two species of *Notiospathius* from Brazil originally described in early twentieth century are redescribed, *Notiospathius caudatus* (Szépligeti) and *Notiospathius diversus* (Szépligeti). Five new species of *Notiospathius* from southern Brazil are also described: *Notiospathius atra* **sp. n.**, *Notiospathius johnlennoni* **sp. n.**, *Notiospathius novateutoniae* **sp. n.**, *Notiospathius sulcatus* **sp. n.**, and *Notiospathius xanthofasciatus* **sp. n.** Most of the type specimens of the above new species were collected in the mid twentieth century in the Nova Teutonia region, which is now part of the municipality of Seara in the state of Santa Catarina.

## Introduction

The braconid subfamily Doryctinae represents one of the most speciose subfamilies of braconid parasitic wasps, with species distributed on all continents but being especially diverse in the tropics (Belokobylskij 1992; Belokobylskij et al. 2004a, b; Marsh 2002). In the Neotropical Region, *Notiospathius* Matthews and Marsh potentially represents the second most diverse doryctine genus, only after the cosmopolitan *Heterospilus*. Its extraordinary species richness, however, has largely been overlooked, with only 27 species described to date (Zaldívar-Riverón and De Jesús-Bonilla 2010, 2011). *Notiospathius* was erected by Matthews and Marsh (1973) to contain 14 species from Central and South America that were originally placed in the mainly Holarctic and Oriental *Spathius*. Since then, only 15 additional species of *Notiospathius* have been described, all of them from Costa Rica (Marsh, 2002), whereas two of the species that were transferred in the description of the genus, *Notiospathius meliorator* (Fabricius) and *Notiospathius necator* (Fabricius), were found to belong to an undescribed doryctine genus that is similar to *Ptesimogaster* (Zaldívar-Riverón and De Jesús-Bonilla 2010).

Recent molecular phylogenetic (Zaldívar-Riverón et al. 2007, 2008) studies suggested the paraphyletic nature of *Notiospathius* with respect to three small Neotropical doryctine genera: *Hansonorum* Marsh, *Masonius* Marsh, and *Tarasco* Marsh. Species of the above four genera are morphologically similar, all having different degrees of enlargement of the basal sternal plate of the first metasomal tergum (acrosternite *sensu* Belokobylskij, 1992). Species of *Masonius* and *Tarasco* can be mainly distinguished from those of *Notiospathius* and *Hansonorum* by absence of the fore wing vein r-m, lack of hind wing vein cu-a (only *Masonius*), and a face swollen between antennae and clypeus (only *Tarasco*) (Marsh, 1993). In contrast, species of *Hansonorum*, are only distinguished from those of *Notiospathius* by the presence of a basal tubercle on the hind coxa (Marsh, 2002), this being one of the features traditionally employed to separate supraspecific taxa in Doryctinae. The relationships recovered by the aforementioned molecular phylogenetic studies revealed that this tubercle was gained and lost on several occasions within the subfamily. Moreover, as confirmed in other doryctine genera (e.g. *Ptesimogaster* Marsh; Zaldívar-Riverón et al. 2010), recent molecular work has revealed that this morphological feature varies among closely related species or even intraspecifically in *Notiospathius*/*Hansonorum* (Ceccarelli et al. submitted).

Currently, only three described species assigned to *Notiospathius* have been recorded for Brazil despite the actual enormous richness of the genus in this country (Nunes, unpubl.): *Notiospathius caudatus* (Szépligeti), *Notiospathius diversus* (Szépligeti), and *Notiospathius leucacrocera* (Enderlein). In this work we describe five new species of *Notiospathius* from southern Brazil, considering *Hansonorum* to be a junior synonym of *Notiospathius*syn. n. [*Notiospathius carolinae* (Marsh) comb. n., *Notiospathius pauli* (Marsh) comb. n.] based on the above molecular phylogenetic evidence. We also redescribe *Notiospathius caudatus* (Szépligeti) and *Notiospathius diversus* based on their holotypes, which were originally described more than a century ago (Szépligeti 1902). We decided to maintain the generic status of *Masonius* and *Tarasco* until additional molecular and morphological information help us to confirm whether they should be synonymised with *Notiospathius*. Most of the type specimens belonging to the new species of *Notiospathius* described in this work were collected during the mid twentieth century in the municipality of Seara, formerly known as Nova Teutonia, in the state of Santa Catarina, by the German entomologist Fritz Plaumann. This region, which was originally composed of mainly Atlantic forest (Mata Atlântica), has been subject to intense deforestation over the last two decades (Baptista 2008), illustrating the urgency for describing its highly overlooked biodiversity.

## Methods

This study was based on material deposited in the following collections: The Natural History Museum, London, UK (NHML), Departamento de Ecologia e Biologia Evolutiva, Universidade Federal de São Carlos, São Carlos, SP, Brazil (DCBU), Colección Nacional de Insectos, Instituto de Biología, Universidad Nacional Autónoma de México (CNIN-UNAM), Hungarian Natural History Museum (HNHM) and Canadian National Collection of Insects, Ottawa, Canada (CNCI). The surface sculpture and wing venation terminologies employed follow Marsh (2002). Colour digital photographs were taken and edited with a Leica® Z16 APO-A stereoscopic microscope, a Leica® DFC295/DFC290 HD camera, and the Leica Application Suite® program. Digital SEM photographs were taken with a FEI QuantaTM 250 SEM in low vacuum mode. Specimens assigned to the five new species described below were compared with type specimens belonging to most of the described species of *Notiospathius* (= *Hansonorum* syn. n.).

## Taxonomy

### Notiospathius atra

De Jesús-Bonilla, Nunes, Penteado-Dias, Zaldívar-Riverón sp. n.

urn:lsid:zoobank.org:act:38C6BAEC-2C24-40CF-89F7-766D8DEBB3D7

http://species-id.net/wiki/Notiospathius_atra

Figs 1A–F 

#### Diagnosis.

**Figure 1.**
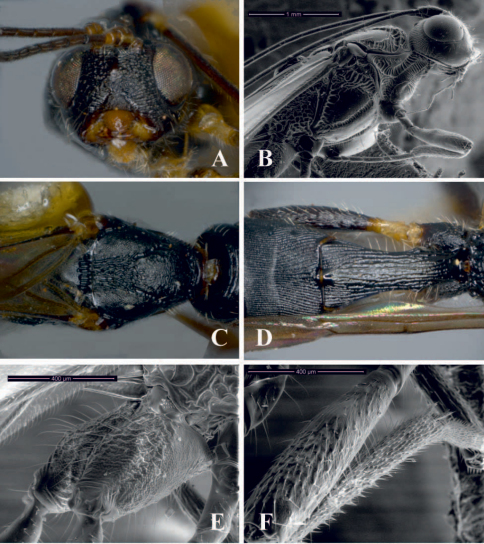
*Notiospathius atra* sp. n.: **A** head, frontal view **B** mesosoma and head, lateral view **C** mesoscutum, dorsal view **D** metasomal median tergites 1-3, dorsal view **E** hind coxa lateral view **F** middle leg with row of spines.

This speciesdiffers from other described Brazilian species of *Notiospathius* by having the following combination of features: (1) fourth median tergite coriaceous basally (smooth in *Notiospathius caudatus*, *Notiospathius diversus*, *Notiospathius leucacrocera* and *Notiospathius novateutoniae* sp. n., smooth to rugose basally in*Notiospathius sulcatus*sp. n., costate on basal half, smooth on apical half in *Notiospathius xanthofasciatus* sp. n., costate basolaterally in *Notiospathius johnlennoni* sp. n.), (2) scutellar disc coriaceous-granulate (Fig. 1C) (coriaceous in *Notiospathius diversus* and *Notiospathius novateutoniae* sp. n., coriaceous-rugose in *Notiospathius xanthofasciatus* sp. n., smooth in *Notiospathius caudatus*, N*. johnlennoni* sp. n., *Notiospathius leucacrocera*, and *Notiospathius sulcatus*sp. n.), and (3) hind coxa with distinct tubercle at base (Fig. 1E) (also present in *Notiospathius diversus*, *Notiospathius novateutoniae* sp. n., and*Notiospathius xanthofasciatus* sp. n.).

#### Description.

Female. *Colour*: Head dark brown to black, pedicel light brown to honey yellow; flagellomeres brown; palpi yellow. Mesosoma and first three metasomal terga black, remaining terga brown except the last one, which is light brown. Ovipositor and sheaths dark brown to black. Fore and middle femora and tibia brown, trochanter and trochantellus yellow, coxae light brown; hind coxa dark brown, trochanter and trochantellus yellow, femur and tibia brown, turning yellow at base; tarsi brown. Wings dusky, veins and stigma brown, tegula dark brown. *Body length*: 5.8 mm (lateral view), ovipositor 6.0 mm. *Head*: Clypeus granulate, face and frons striate-rugose, face with smooth area in the middle, vertex striate to striate-rugose, temple striate, gena smooth (Fig. 1A, B); eye 1.1 times higher than wide (lateral view); malar space 0.4 times eye height (lateral view); temple 0.4 times eye width (dorsal view); hypoclypeal depression elliptic; ocular-ocellar distance 2.4 times diameter of lateral ocellus; length of scape 1.5 times its width (frontal view); antenna with 31 flagellomeres. *Mesosoma*: Length of mesosoma twice its maximum height; pronotum laterally costate-rugose, pronotal groove wide and scrobiculate, propleuron costate on anterior half, finely coriaceous on posterior half; lateral mesoscutal lobes coriaceous, slightly rugose laterally, median mesoscutal lobe coriaceous, rugose posteriorly (Fig. 1C); notauli scrobiculate, meeting before scutellum at middle of mesoscutum in a large, longitudinally rugose area; scutellar disc coriaceous on anterior half, granulate on posterior half (Fig. 1C); mesopleural sulcus surrounding mesopleuron strongly scrobiculate, mesopleuron coriacoeus medially and ventrally (Fig. 1B); precoxal sulcus wide, scrobiculate, as long as mesopleuron; venter of mesosoma coriaceous; propodeum and metapleuron rugose-areolate, propodeum with slightly indicated median longitudinal carina running to basal half; apical lateral corners without distinct tubercles, spines over hind coxa indistinct. *Wings*: Fore wing length 3.8 times its maximum width, length of pterostigma 3.8 times its maximum width, vein r 0.22 length of vein 3RSa, vein m-cu reaching first submarginal cell before vein 2RS, vein 1cu-a distinctly postfurcal to vein 1M; hind wing vein M+CU 0.5 times length of vein 1M. *Legs*: Middle and hind femora coriaceous, hind coxa coriaceous ventrally, slightly coriaceous-rugose dorsally with distinct tubercle at base (Fig. 1E); middle tibia with a row of at least seven spines (Fig. 1F). *Metasoma*: First metasomal median tergite rugose to costate-rugose, length 1.9–2.1 times its apical width (dorsal view) (Fig. 1D); basal sternal plate (acrosternite) about 0.5 times length of tergum; second and third median tergites costate with rugose microsculpture (Fig. 1D); suture between second and third median tergites distinct and sinuate; fourth median tergite coriaceous basolaterally, remaining area smooth and polished; remaining median tergites smooth and polished; ovipositor about 1.9 times length of metasoma.

Male. Unknown.

Variation. Females: *Body length*: 4.7–5.8 mm (lateral view), ovipositor 4.7–6.0 mm. *Head:* Eye 1.0–1.2 times higher than wide (lateral view); malar space 0.3–0.5 times eye height (lateral view); antenna with 27–33 flagellomeres. *Wings*: Fore wing length 3.5–4.0 times its maximum width, length of pterostigma 3.6–3.8 times its maximum width. *Metasoma*: length of first metasomal median tergite 1.9–2.1 times its apical width (dorsal view); ovipositor about 1.9–2.0 times length of metasoma.

#### Holotype.

Female (NHML). “Brasil, Nova Teutonia, 27°11'S, 52°23'W; 22-XI-1940; Fritz Plaumann coll, B. M. 1957-341”.

#### Paratypes.

Four females (NHML, CNIN-UNAM). Same data as holotype.

#### Biology.

Unknown.

#### Etymology.

From the Latin *atra*, meaning dark or black, due to the dark body colour of the species.

### Notiospathius caudatus

(Szépligeti)

http://species-id.net/wiki/Notiospathius_caudatus

Figs 2A, B

Psenobolus caudatus Szépligeti, 1902
Notiospathius caudatus Matthews & Marsh, 1973

#### Diagnosis.

**Figure 2.**
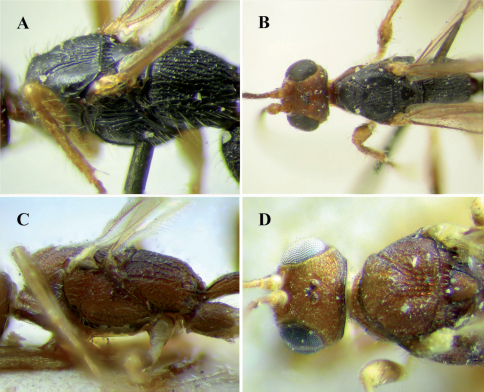
*Notiospathius caudatus*: **A** mesosoma, lateral view **B** Head and mesosoma, dorsal view; *Notiospathius diversus*: **C** mesosoma, lateral view **D** head and mesoscutum, dorsal view.

This species differs from the remaining described Brazilian species of *Notiospathius* by having the following combination of features: (1) vertex striate (striate in *Notiospathius johnlennoni* sp. n.and *Notiospathius leucacrocera*, striate to striate-rugose in *Notiospathius atra*, rugose or striate-rugose in *Notiospathius diversus*, *Notiospathius novateutoniae* sp. n., *Notiospathius sulcatus* sp. n. and *Notiospathius xanthofasciatus* sp. n.), (2) scutellar disc smooth (Fig. 2B) (see *Notiospathius atra* diagnosis for character states of remaining species), (3) mesoscutal lobes coriaceous, transversally costate laterally (completely coriaceous in *Notiospathius leucacrocera*, with rugose areas in *Notiospathius atra*, *Notiospathius diversus*, *Notiospathius johnlennoni* sp. n., *Notiospathius novateutoniae* sp. n., *Notiospathius sulcatus* sp. n. and *Notiospathius xanthofasciatus* sp. n.), and (4) three first metasomal median tergites sculptured (only first two metasomal median tergites sculptured in *Notiospathius diversus*).

#### Description.

Female. *Colour*: Head brown, scape and pedicel light brown; flagellomeres brown; palpi yellow. Mesosoma and first metasomal tergum black, remaining terga brown. Ovipositor and sheaths brown. Fore and middle coxae and basal third of femora light brown, trochanter and trochantellus pale yellow, apical two thirds of femora, tibiae and tarsi brown; hind coxa dark brown to black, trochanter and trochantellus pale yellow, femur brown, basal third of tibia light brown, turning brown apically, tarsi brown. Wings dusky, veins and stigma brown, tegula brown. *Body length*: 5.1 mm (lateral view), ovipositor 5.4 mm. *Head*: Clypeus transversally costate, face striate-rugose, frons and vertex striate, temple smooth, gena smooth; eye 1.2 times higher than wide (lateral view); malar space 0.4 times eye height (lateral view); temple 0.4 times eye width (dorsal view); hypoclypeal depression elliptic; ocular-ocellar distance 2.2 times diameter of lateral ocellus; length of scape 1.3 times its width (frontal view); antennae broken, 18–20 flagellomeres remaining. *Mesosoma*: Length of mesosoma 1.8 times its maximum height; pronotum laterally costate, pronotal groove wide and scrobiculate, propleuron costate on anterior half, smooth on posterior half; mesoscutal lobes coriaceous, transversally costate laterally, median mesoscutal lobe costate-rugose posteriorly (Fig. 2B); notauli wide, deep and scrobiculate, not meeting before scutellum, finishing in a large longitudinally costate-rugose area; scutellar disc smooth (Fig. 2B); mesopleuron porcate dorsally, coriaceous-rugose medially and ventrally; precoxal sulcus wide, scrobiculate, as long as mesopleuron (Fig. 2A); venter of mesosoma slightly coriaceous; propodeum and metapleuron rugose, with a series of longitudinal carinae (Fig. 2B), apical lateral corners without distinct tubercles, spines over hind coxa indistinct. *Wings*: Fore wing length 4.0 times its maximum width, length of pterostigma 4.0 times its maximum width, vein r 0.2 times length of vein 3RSa, vein m-cu interstitial to vein 2RS, vein 1cu-a interstitial to vein 1M; hind wing vein M +CU 0.4 times length of vein 1M. *Legs*: Middle and hind femora smooth to slightly coriaceous, hind coxa coriaceous ventrally, costate dorsally, without distinct tubercle at base; middle tibia with a row of at least six spines. *Metasoma*: First metasomal median tergite costate-rugose with coriaceous microsculpture; length 3.2 times its apical width (dorsal view); basal sternal plate (acrosternite) about 0.6 times length of tergum; second median tergite costate with rugose microsculpture; suture between second and third median tergites distinct and not sinuate; third median tergite costate with rugose microsculpture basally, remaining area smooth and polished; remaining median tergites smooth and polished; ovipositor about 2.0 times length of metasoma.

Male. Unknown.

#### Holotype.

Female **(**HNHM). **“**Fonteboa, Brasil. Hym. Typ. No. 1605, Museum Budapest.”

#### Biology.

Unknown.

### Notiospathius diversus

(Szépligeti)

http://species-id.net/wiki/Notiospathius_diversus

Figs 2C, D

Spathius diversus Szépligeti, 1902
Notiospathius diversus Matthews & Marsh, 1973

#### Diagnosis.

This species differs from the remaining described species of *Notiospathius* by having the following combination of features: (1) vertex striate-rugose (Fig. 2D) (see *Notiospathius caudatus* diagnosis for character states of remaining species), (2) scutellar disc coriaceous (Fig. 2D) (see *Notiospathius atra* diagnosis for character states of remaining species), (3) mesoscutal lobes coriaceous, with large rugoe areas medially and laterally (see *Notiospathius caudatus* diagnosis for character states of remaining species), and (4) third metasomal median tergite smooth (scultpured in the remaining species).

**Description.** Female. *Colour*: Head brown, pedicel and scape light brown; flagellomeres light brown, turning brown to apex; palpi yellow. Mesosoma and first two metasomal terga brown, remaining terga dark brown. Ovipositor and sheaths yellow, turning brown to apex. Fore and middle coxae, trochanter and trochantellus yellow, femora, tibiae and tarsi light brown; hind coxa brown, trochanter, trochantellus and apical third of tibia yellow, basal two thirds of femur brown, tibia and tarsi light brown. Wings dusky, veins and stigma brown, tegula yellow. *Body length*: 3.8 mm *(*lateral view), ovipositor 2.3 mm. *Head*: Clypeus coriaceous, face, frons and vertex striate-rugose, temple striate, gena smooth; eye 1.2 times higher than wide (lateral view); malar space 0.4 times eye height (lateral view); temple 0.5 times eye width (dorsal view); hypoclypeal depression elliptic; ocular-ocellar distance three times diameter of lateral ocellus; length of scape 1.8 times its width (frontal view); antennae broken, 1–17 flagellomeres remaining. *Mesosoma*: Length of mesosoma 1.9 times its maximum height; pronotum laterally costate-rugose, pronotal groove wide and scrobiculate, propleuron costate; lateral mesoscutal lobes coriaceous, with large rugose areas medially and laterally, median mesoscutal lobe coriacoeus-rugose anteriorly, strongly rugose posteriorly (Fig. 2D); notauli scrobiculate, turning smooth on posterior third, not meeting before scutellum, finishing in a large longitudinally costate-rugose area (Fig. 2D); scutellar disc coriaceous; mesopleuron porcate-rugose dorsally, coriaceous-slightly rugose medially and ventrally (Fig. 2C); precoxal sulcus wide, scrobiculate, as long as mesopleuron; venter of mesosoma coriaceous; propodeum and metapleuron rugose-areolate, propodeum with a median longitudinal carina running to basal half; apical lateral corners without distinct tubercles, spines over hind coxa indistinct. *Wings*: Fore wing length 3.5 times its maximum width, length of pterostigma 4.0 times its maximum width, vein r 0.4 times length of vein 3RSa, vein m-cu postfurcal to vein 2RS, vein 1cu-a distinctly postfurcal to vein 1M; hind wing vein M +CU about 0.5 times length of vein 1M. *Legs*: Middle and hind femora slightly coriaceous, hind coxa coriaceous dorsally, costate ventrally, with a distinct tubercle at base; spines on middle tibia not visible due to leg position. *Metasoma*: First metasomal median tergite rugose basally, turning costate with coriaceous microsculpture costate medially and apically, length 2.0 times its apical width (dorsal view); basal sternal plate (acrosternite) about 0.5 times length of tergum; second median tergite slightly costate-coriaceous anteriorly, remaining area smooth; suture between second and third median tergites distinct and slightly sinuate; remaining median tergites smoothand polished; ovipositor about 0.9 times length of metasoma.

Male. Unknown.

#### Holotype.

Female **(**HNHM).“Brasilien, Blumenau, 738-47, Hym. Typ. No. 1604.”

#### Biology.

Unknown.

### Notiospathius johnlennoni

De Jesús-Bonilla, Nunes, Penteado-Dias, Zaldívar-Riverón sp. n.

urn:lsid:zoobank.org:act:FF0548AB-A220-475D-AEB8-3FB259163C1F

http://species-id.net/wiki/Notiospathius_johnlennoni

Figs 3A–F 

#### Diagnosis.

**Figure 3.**
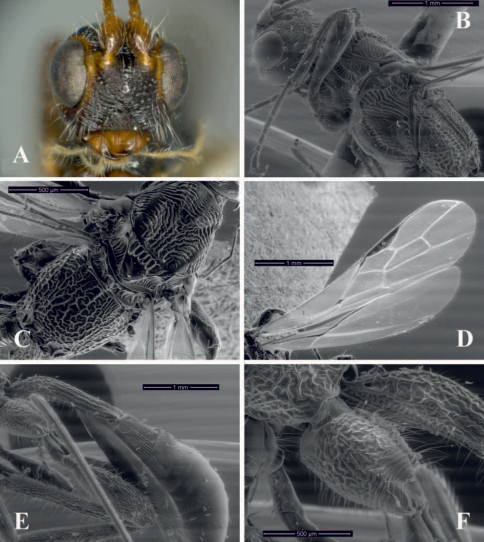
*Notiospathius johnlennoni* sp. n.: **A** head, frontal view **B** mesosoma and head, lateral view **C** mesosoma, dorsal view **D** fore and hind wings **E** metasoma, lateral view **F** hind coxa, lateral view.

This speciesdiffers from the remaining described Brazilian species of *Notiospathius* by having the following combination of features: (1) most flagellomeres bicoloured, brown on basal half, turning honey yellow apically (flagellomeres having one colour in the remaining species), (2) fourth median tergite costate basolaterally (Fig. 3E) (see *Notiospathius atra* diagnosis for character states of remaining species), and (3) fifth median tergite usually with striate microsculpture basolaterally (Fig. 3E) (smooth and polished in the remaining species).

#### Description.

Female. *Colour*: Head brown to light brown, eye orbits honey yellow; scape brown to light brown, pedicel light brown; first flagellomere light brown, following flagellomeres bicoloured, brown on basal half, turning honey yellow apically, apical eight flagellomeres yellow; palpi yellow. Mesosoma and first metasomal tergum dark brown to black, remaining terga brown. Ovipositor and sheaths light brown, dark brown at apex. Fore and middle coxae and trochantellus light brown to brown, femora and tibiae brown; hind coxa dark brown to black, trochanter and trochantellus light brown, femur brown, tibia brown basally, turning yellow apically; tarsi brown. Wings dusky, stigma and veins brown, tegula yellow to light brown. *Body length*: 6.5 mm (lateral view), ovipositor 7.0 mm. *Head*: Clypeus granulate-rugose, face and frons striate-rugose, vertex striate to striate-rugose, temple striate, gena smooth (Fig. 3A); eye 1.5 times higher than wide (lateral view); malar space 0.4 times eye height (lateral view); temple 0.6 times eye width (dorsal view); hypoclypeal depression elliptic; ocular-ocellar distance 2.4 times diameter of lateral ocellus; length of scape 1.6 times its width (frontal view); antenna with 29 flagellomeres. *Mesosoma*: Length of mesosoma twice its maximum height; pronotum laterally costate-rugose, pronotal groove strongly scrobiculate, propleuron costate; mesoscutal lobes transversally costate-rugose laterally, slightly coriaceous medially (Fig. 3C); notauli scrobiculate anteriorly, meeting before scutellum at middle of mesoscutum in a large costate-rugose area (Fig. 3C); scutellar disc smooth; mesopleuron porcate dorsally, smooth-slightly rugose medially and ventrally (Fig. 3B); precoxal sulcus wide, scrobiculate, as long as mesopleuron; venter of mesosoma slightly rugose; propodeum and metapleuron rugose-areolate, propodeum with a slightly indicated median longitudinal carina running to basal half; apical lateral corners without distinguishable tubercles, spines over hind coxae short and blunt. *Wings*: Fore wing length 3.8 times its maximum width, length of pterostigma 5.0 times its maximum width, vein r about 0.2 times length of vein 3RSa, vein m-cu interstitial or slightly postfurcal to vein 2RS, vein 1cu-a interstitial with vein 1M (Fig. 3D); hind wing vein M+CU 0.4–0.45 length of vein 1M. *Legs*: Fore and middle femora rugose dorsally, hind femur costate-rugose dorsally, slightly rugose-coriaceous ventrally; hind tibia densely pilose, middle tibia with a row of at least four spines; hind coxa without tooth or tubercle at base (Fig. 3F). *Metasoma*: First metasomal median tergite rugose basally, turning costate-rugose apically, length 2.6–3.1 times its apical width (dorsal view); basal sternal plate (acrosternite) about 0.7 times length of tergum; second median tergite costate with rugose microsculpture; suture between second and third median tergites poorly defined and straight dorsally; third median tergite costate (Fig. 3E); fourth median tergite costate basolaterally, remaining area smooth and polished; fifth median tergite usually with striate microsculpture basolaterally; remaining median tergites smooth and polished; ovipositor about two times length of metasoma.

Male. Smaller than female. Body length 3.8 mm.

Variation. Female. *Colour*: apical 3-8 flagellomeres yellow. *Body length*: 5.3–7.0 mm (lateral view), ovipositor 5.8–9 mm. *Head*: Eye 1.3–1.6 times higher than wide (lateral view); malar space 0.4–0.5 times eye height (lateral view); ocular-ocellar distance 2.0–2.6 times diameter of lateral ocellus; length of scape 1.5–1.8 times its width (frontal view); antenna with 25–36 flagellomeres. *Wings*: Fore wing length 3.8–4.0 times its maximum width, length of pterostigma 4.8–5.0 times its maximum width. *Metasoma*: Length of first metasomal median tergite 2.8 times its apical width (dorsal view).

#### Holotype.

Female (NHML). “Brasil, Nova Teutonia, 27°11'S, 52°23'W; 3-XI-1938; Fritz Plaumann coll, B. M. 1938-632”.

#### Paratypes.

Fourteen specimens, 10 females, four males. One female (DCBU), “Jundiai do Sul, PR, Brasil, Faz. Monte Verde, Lev. Ent. PROFAUPAR, 11/1/1998, armadilha Malaise”; one female (CNCI), “Brasil, Nova Teutonia, 27°11'S, 52°23'W; 21-II-1960; Fritz Plaumann coll.”; remaining specimens (NHML, CNIN-UNAM) with same data as holotype.

#### Biology.

Unknown.

#### Etymology.

This species is named in honour of the 30th anniversary of the death of the British musician John Lennon in 2010.

### Notiospathius novateutoniae

De Jesús-Bonilla, Nunes, Penteado-Dias, Zaldívar-Riverón sp. n.

urn:lsid:zoobank.org:act:869C46BC-61D9-40AC-B8E7-220E22D065ED

http://species-id.net/wiki/Notiospathius_novateutoniae

Figs 4A–H 

#### Diagnosis.

**Figure 4.**
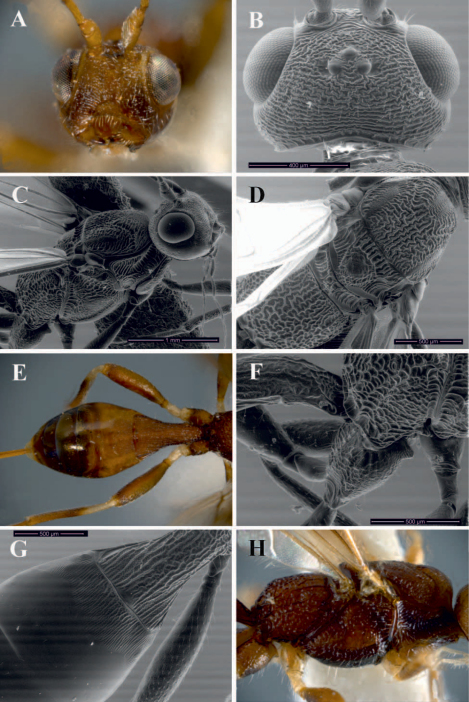
*Notiospathius novateutoniae* sp. n.: **A** head, frontal view **B** head, dorsal view **C** mesosoma and head, lateral view **D** mesosoma, dorsal view **E** metasoma, dorsal view **F** hind coxa, lateral view **G** metasomal median tergites 1-3, dorsal view **H** mesosoma, lateral view.

This species differs from the remaining Brazilian species of *Notiospathius* by having: (1) suture between second and third median tergites strongly sinuate, with two lateral, subparallel depressions (Fig. 4E) (not sinuate and without subparallel depressions in the remaining species), (2) fourth median tergite costate on basal half, smooth on apical half (see *Notiospathius atra* diagnosis for character states of remaining species), and (3) hind coxa with a distinct tubercle at base (Fig. 4F) (see *Notiospathius atra* diagnosis for character states of remaining species).

#### Description.

Female. *Colour*: Head brown to light brown, scape and pedicel light brown to honey yellow; flagellomeres light brown, turning brown to apex; palpi honey yellow to white. Mesosoma and first metasomal tergum brown (Fig. 4E, H), second to fourth terga light brown to honey yellow, remaining terga brown except the last one, which is honey yellow. Ovipositor and sheaths light brown, dark brown at apex. Fore and middle coxae, trochanter, trochantellus, tibiae and tarsi pale yellow; all femora brown to light brown; hind coxa and tarsus brown to light brown, hind tibia brown to light brown with pale yellow base. Wings slightly dusky, veins brown, stigma brown with yellow base, tegula honey yellow. *Body length*: 6.0 mm (lateral view), ovipositor 5.0 mm. *Head*: Clypeus granulate-rugose, face and frons striate-rugose, vertex striate-rugose to rugose near ocelli, temple striate, gena smooth (Fig. 4A, B); eye 1.3 times higher than wide (lateral view); malar space 0.6 times eye height (lateral view); temple 0.7 times eye width (dorsal view); hypoclypeal depression elliptic; length of scape 1.5 times its width (frontal view); antenna with 28 flagellomeres. *Mesosoma*: Length of mesosoma around 1.8 times its maximum height; pronotum laterally costate-rugose, pronotal groove strongly scrobiculate, propleuron costate-rugose; mesoscutal lobes coriaceous medially, transversally costate-rugose laterally; notauli scrobiculate anteriorly, not joining, interrupting before scutellum at middle of mesoscutum in a large costate-rugose to rugose area (Fig. 4D); scutellar disc coriaceous; mesopleuron porcate-rugose dorsally, coriaceous medially and ventrally (Fig. 4C, H); precoxal sulcus wide, scrobiculate, as long as mesopleuron; venter of mesosoma slightly rugose-coriaceous; propodeum and metapleuron rugose-areolate, propodeum without distinct longitudinal carina or areola, without propodeal spines or tubercles. *Wings*: Fore wing length 3.7 its maximum width, length of pterostigma 3.6 times its maximum width, vein r about 0.3 length of vein 3RSa, vein m-cu interstitial or slightly basal to vein 2RS, vein 1cu-a postfurcal to vein 1M; hind wing vein M+CU 0.6 length of vein 1M. *Legs*: Hind coxa rugose dorsally, coriaceous ventrally, with a well-defined tubercle at base (Fig. 4F); tibiae and femora coriaceous, middle tibia with a row of at least seven spines. *Metasoma*: First metasomal median tergite costate apically, rugose basally, with coriaceous microsculpture, length 1.6 times its apical width (dorsal view) (Fig. 4G); basal sternal plate (acrosternite) 0.6 times length of tergum; second median tergite costate with coriaceous microsculpture medially; suture between second and third median tergites strongly sinuate, with two lateral, subparallel depressions (Fig. 4G); suture between third and fourth median tergites indistinct; remaining median tergites smooth and polished; ovipositor about 1.6 times length of metasoma.

Male. Smaller than female. Body length 3.0–3.6 mm.

Variation. Females. *Body length*: 4.3–6.0 mm (lateral view), ovipositor 3.7–5.0 mm. *Head*: Eye 1.1–1.3 times higher than wide (lateral view); malar space 0.5–0.6 times eye height (lateral view); antenna with 23–28 flagellomeres. *Wings*: Fore wing length 3.6–3.7 its maximum width. *Metasoma*: Length of first metasomal median tergite 1.5–1.6 times its apical width (dorsal view); basal sternal plate (acrosternite) 0.5–0.6 times length of tergum.

#### Holotype.

Female (NHML). “Brasil, Nova Teutonia, 27°11'S, 52°23'W; 16-VIII-1944; Fritz Plaumann coll, B. M. 1938-632”.

#### Paratypes.

Ninety specimens, 85 females, 10 males. (NHML, CNIN-UNAM). Same data as holotype.

#### Biology.

Unknown.

#### Etymology.

The name *novateutoniae* refers the previous name of the type locality of this and all species described in this study, Nova Teutonia. This municipality is currently named as Seara and is located in the state of Santa Catarina, in the south of Brazil.

### Notiospathius sulcatus

De Jesús-Bonilla, Nunes, Penteado-Dias, Zaldívar-Riverón sp. n.

urn:lsid:zoobank.org:act:DB5A6568-FACB-4545-8CEC-0AA249AD1C7D

http://species-id.net/wiki/Notiospathius_sulcatus

Figs 5A–D 

#### Diagnosis.

**Figure 5.**
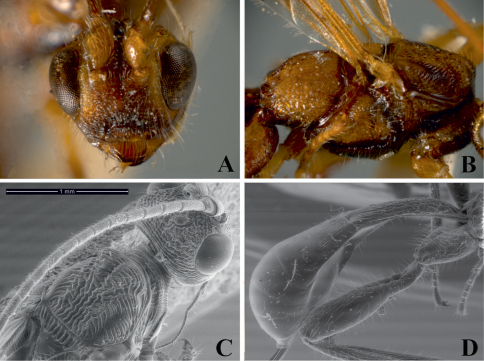
*Notiospathius sulcatus* sp. n.: **A** head, frontal view **B** mesosoma, lateral view **C** mesoscutum and head, dorsal view **D** metasoma, lateral view.

This species differs from the remaining described Brazilian species of *Notiospathius* by having the following combination of features: (1) median mesoscutal lobe with a deep longitudinal groove running medially (Fig. 5C) (absent in the remaining species), (2) mesopleuron smooth medially and ventrally (Fig. 5B) (coriaceous in *Notiospathius atra* and *Notiospathius novateutoniae*, rugose-coriaceous in *Notiospathius xanthofasciatus* sp. n., coriaceous-rugose in *Notiospathius caudatus*, coriaceous-rugose in *Notiospathius diversus*, smooth-rugose in *Notiospathius johnlennoni* and *Notiospathius leucacrocera*), (3) venter of mesopleuron and venter of propodeum dark brown to black, contrasting with the light brown colour of the remainder of the mesosoma (Fig. 5B) (with different coloration in the remaining species), and (4) face, frons and vertex strongly rugose or striate-rugose (Fig. 5A) (not strongly rugose in the remaining species).

#### Description.

Female. *Colour*: Head brown, orbit surrounding eyes light brown; scape light brown, with a longitudinal brown stripe laterally, pedicel brown; first flagellomere brown, following flagellomeres light brown, turning brown at apex, seven apical flagellomeres yellow; palpi white to pale yellow. Mesosoma light brown; propleuron and pronotal groove region brown to dark brown; lateral mesoscutal lobes brown medially; venter of mesopleuron and venter of propodeum dark brown to black. First metasomal tergum light brown to brown, remaining terga light brown to pale yellow, with sutures between median tergites brown. Ovipositor and sheaths honey yellow to light brown, dark brown to black at apex. Legs honey yellow to brown, usually with fore and middle coxae, trochanter and trochantellus lighter. Wings slightly dusky, stigma,veins and tegula light brown to honey yellow. *Body length*: 6.5 mm (lateral view), ovipositor 7.2 mm. *Head*: Clypeus granulate-rugose, face striate-rugose, frons and vertex rugose to striate-rugose, temple striate, gena smooth (Fig. 5A); eye 1.4 times higher than wide (lateral view); malar space 0.5 times eye height (lateral view); temple 0.5 times eye width (dorsal view); hypoclypeal depression elliptic; ocular-ocellar distance 3.2 times diameter of lateral ocellus; length of scape 1.7 times its width (frontal view); antenna with 34 flagellomeres. *Mesosoma*: Length of mesosoma twice its maximum height; pronotum laterally costate to costate-rugose, pronotal groove smooth to weakly scrobiculate, propleuron costate anteriorly, smooth posteriorly; mesoscutal lobes transversally costate to costate-rugose, median mesoscutal lobe costate-coriaceous medially, with a deep longitudinal groove running medially; notauli deep and scrobiculate, meeting before scutellum at middle of mesoscutum in a large costate-rugose area (Fig. 5C); scutellar disc smooth; mesopleuron porcate dorsally, smooth medially and ventrally, slightly costate-rugose antero-ventrally (Fig. 5B); precoxal sulcus wide, scrobiculate, as long as mesopleuron; venter of mesosoma smooth; propodeum and metapleuron entirely rugose, without visible median carina or areola; apical lateral corners without distinguishable tubercles, spines over hind coxae short and slightly pointed. *Wings*: Fore wing length 3.5 times its maximum width, length of pterostigma 4.7 times its maximum width, vein r about 0.2 length of vein 3RSa, vein m-cu interstitial with vein 2RS, vein 1cu-a slightly to distinctly postfurcal to vein 1M; hind wing vein M+CU 0.5 length of vein 1M. *Legs*: Hind coxa rugose ventrally, costate dorsally without tooth or tubercle at base; middle tibia with a row of at least seven spines. *Metasoma*: First metasomal median tergite rugose basally, turning costate-rugose apically, length around 3.2 times its apical width (lateral view) (Fig. 5D); basal sternal plate (acrosternite) about 0.6 times length of tergum; second median tergite costate with rugose microsculpture (Fig. 5D); third median tergite finelly costate; suture between second and third median tergites weakly sinuate; suture between third and fourth median tergites almost indistinct; remaining median tergites smooth and polished; ovipositor 2.5 times length of metasoma.

Male. Smaller than female. Fourth metasomal median tergite rugose basally, mesosoma of some specimens slightly darker than females; suture between third and fourth median tergites considerably curved to base.

Variation. Females. *Colour*: seven to 10 apical flagellomeres yellow. *Body length*: 6.0–8.0 mm (lateral view), ovipositor 5.2–10 mm. *Head*: Eye 1.3–1.4 times higher than wide (lateral view); malar space 0.4–0.6 times eye height (lateral view); ocular-ocellar distance 3.0–3.8 times diameter of lateral ocellus; antenna with 30–38 flagellomeres. *Wings*: Fore wing length 3.0–3.9 times its maximum width, length of pterostigma 4.2–5.0 times its maximum width. *Metasoma*: ovipositor 2.3–2.5 times length of metasoma.

#### Holotype.

Female (NHML). “Brasil, Nova Teutonia, 27°11'S, 52°23'W; 4-V-1938; Fritz Plaumann coll, B. M. 1938-682”.

#### Paratypes.

Twenty one specimens, 12 females, nine males. One female (CNCI), “Brazil, Est. Rio de Janeiro, Silva Jardim, III.1974, F. M. Oliveira col.”; one female (DCBU), “BIOTA-FAPESP, Nova Iguaçú, RJ, Brasil, Reserva Biológica do Tinguá, 6-9.III.2002, Moericke, 5ª trilha, S.T.P. Amarante col.”; two females (DCBU), “BIOTA-FAPESP, Sta. Maria Madalena, RJ, Brasil, Parque Estadual do Desengano, 16-19.IV.2002, 560m, Moericke, 2ª Trilha Bosque, Penteado-Dias col.”, and “20-23.IV.2002, 2ª Bosque, Penteado-Dias col.”; two females, one male (DCBU) “BIOTA-FAPESP, Santa Tereza, ES, Brasil, Est. Biol. Sta. Lúcia, 749, 755, and 867 m, respectively, 9-12.IV.2001, Moericke, ponto T2, C.O. Azevedo & equip col.”; one female (DCBU), “BIOTA-FAPESP, Pque. Est. Intervales, SP, Brasil, Base Barra Grande, Trilha da Anta, 11-14.XII.2000, Moericke, Ponto B9, M.T. Tavares e equioe col.”; one female (DCBU), “Ubatuba, SP, Brasil, 29.I.1990, N.F. Cristo col.”; one female (DCBU), “BIOTA-FAPESP, Morretes, PR, Brasil, Parque Est. do Pau Oco, 11-14.IV.2002, Moericke, Ponto 6 Bosque, M.T. Tavares e equipe col.”; one male (DCBU), “BIOTA-FAPESP, Nova Iguaçú, RJ, Brasil, Reserva Biológica do Tinguá, Varredura, Ponto 13, 8.III.2002, S.T.P. Amarante col.”; one male (DCBU), “BIOTA-FAPESP, Sta. Maria Madalena, RJ, Brasil, Parque Estadual do Desengano, 20.IV.2002, 560m, Varredura, 15:32 a 15:37, Penteado-Dias col.”; five males (DCBU), “BIOTA-FAPESP, Santa Tereza, ES, Brasil, Est. Biol. Sta. Lúcia, 755m, 7.IV.2001, Varredura Pto. 7; 8.IV.2001 Varredura Pto. 21; 867m 11.IV.2001, Varredura Pto. 48 (two of these males with 867m 11.IV.2001, Varredura pto 44, C.O. Azevedo & equip col.)”; one male (DCBU), “BIOTA-FAPESP, Peruíbe, SP, Brasil, Est. Ecol. Juréia-Itatins, 5.V.2002, Varredura 29, Bosque, N.W. Periotto e equip ecol.”; remaining specimens (NHML, CNIN-UNAM) with same data as holotype.

#### Biology.

Unknown.

#### Etymology.

From the perfect passive infinitive Latin word *sulco*, referring to the deep longitudinal groove that runs along the median mesoscutal lobe in this species.

### Notiospathius xanthofasciatus

De Jesús-Bonilla, Nunes, Penteado-Dias, Zaldívar-Riverón sp. n.

urn:lsid:zoobank.org:act:DB5A6568-FACB-4545-8CEC-0AA249AD1C7D

http://species-id.net/wiki/Notiospathius_xanthofasciatus

Figs 6A–D 

#### Diagnosis.

**Figure 6.**
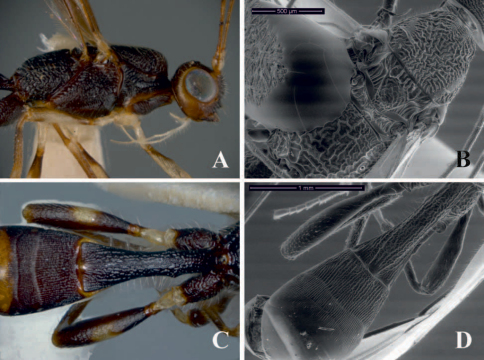
*Notiospathius xanthofasciatus* sp. n.: **A** mesosoma and head, lateral view **B** mesosoma, dorsal view **C** metasomal median tergites 1-3, dorsal view **D** metasoma, dorsal view.

This species differs from the remaining described Brazilian species of *Notiospathius* by having the following combination of features: (1) hind femur brown with yellow transverse stripe in the middle (Fig. 6C) (hind femur without yellow transverse stripe in the remaining species), (2) fourth metasomal median tergite sculptured on basal half (Fig. 6D) (see *Notiospathius atra* diagnosis for character states of remaining species), (3) mesopleuron rugose dorsally (Fig. 6A) (at least partially porcate or coriaceous dorsally in the remaining species), and (4) hind coxa with a distinct tubercle at base (see *Notiospathius atra* diagnosis for character states of remaining species).

#### Description.

Female. *Colour*: Head brown to light brown, eye orbits yellow; scape and pedicel honey yellow; flagellomeres honey yellow, turning brown at apex; palpi yellow to white. Mesosoma and first metasomal tergum brown to dark brown, remaining terga brown except the last one, which is light brown. Ovipositor and sheaths light brown, dark brown to black at apex. Fore and middle coxae, trochanter and trochantellus yellow, fore femur yellow, turning brown dorsally, middle femur brown with a lighter transversal stripe medially, fore and middle tibiae light brown; hind coxa dark brown to black, trochanter and trochantellus yellow, femur brown with yellow transverse stripe medially, tibia light brown, turning yellow to white apically; tarsi light brown to honey yellow. Wings dusky, veins brown, stigma brown with yellow at extreme base, tegula honey yellow to ligth brown. *Body length*: 5.0 mm, ovipositor 6.0 mm. *Head*: Clypeus granulate, face striate-rugose, frons striate-rugose to rugose, vertex strongly rugose anteriorly, striate-rugose posteriorly, temple striate, gena smooth; eye 1.2 times higher than wide (lateral view); malar space 0.5 times eye height (lateral view); temple 0.3 times eye width (dorsal view); hypoclypeal depression elliptic; ocular-ocellar distance 2.3–2.6 times diameter of lateral ocellus; length of scape 1.6 times its width (frontal view); antenna with 32 flagellomeres. *Mesosoma*: Length of mesosoma about twice its maximum height; pronotum laterally costate-rugose, pronotal groove wide and scrobiculate, propleuron costate-coriaceous; mesoscutal lobes strongly rugose, with a median, transverse coriaceous area (Fig. 6B); notauli scrobiculate, meeting before scutellum at middle of mesoscutum in a large rugose area (Fig. 6B); scutellar disc coriaceous- rugose; mesopleuron rugose dorsally, rugose-coriaceous medially and ventrally (Fig. 6A); precoxal sulcus wide, scrobiculate, as long as mesopleuron; venter of mesosoma coriaceous, region near precoxal sulcus slightly transversely striate; propodeum and metapleuron rugose, propodeum without median longitudinal carina or areola; apical lateral corners without tubercles, spines over hind coxae absent. *Wings*: Fore wing length 4.1 times its maximum width, length of pterostigma 5.3 times its maximum width, vein r 0.2 times length of vein 3RSa, vein m-cu interstitial with vein 2RS, vein 1cu-a interstitial with vein 1M; hind wing vein M+CU 0.6 times length of vein 1M. *Legs*: Hind coxa rugose-coriaceous, with a well-defined tubercle at base; fore, middle and hind tibiae granulate; fore, middle and hind femora coriaceous; middle tibia with a row of at least seven spines. *Metasoma*: First metasomal median tergite rugose basally, costate-rugose apically, length 2.8 times its apical width (lateral view); basal sternal plate (acrosternite) about 0.6 times length of tergum (Fig. 6C, D); second and third median tergites costate with rugose microsculpture, sutures between second and third and third and fourth median tergites distinct and sinuate; fourth median tergite costate on basal half, smooth on apical half (Fig. 6D); remaining median tergites smooth and polished; ovipositor 1.5 times length of metasoma.

Male. Smaller than female. Body length 3.7 mm.

Variation. Female. *Body length*: 4.5–6.0 mm. *Head*: eye 1.1–1.2 times higher than wide (lateral view); malar space 0.4–0.5 times eye height (lateral view); ocular-ocellar distance 2.3–2.6 times diameter of lateral ocellus; length of scape 1.4–1.6 times its width (frontal view); antenna with 30–32 flagellomeres. *Wings*: Length of pterostigma 5.0–5.3 times its maximum width. *Metasoma*: Length of first metasomal median tergite 2.6–2.9 times its apical width (lateral view); ovipositor 1.5–1.6 times length of metasoma.

#### Holotype.

Female (NHML). “Brasil, Nova Teutonia, 27°11'S, 52°23'W; 30-XII-1938; Fritz Plaumann coll, B. M. 1937-724”.

#### Paratypes.

Five females, three males (NHML, CNIN-UNAM). Same data as holotype.

#### Biology.

Unknown.

#### Etymology.

From the Greek *xanthos*, meaning yellow or golden, and the Latin *fascia*, meaning band or stripe, referring to the yellow stripe on the hind femur of the species.

#### Comments.

We examined a large series of specimens from south and southeast Brazil that are morphologically very similar to *Notiospathius xanthofasciatus*. However, the latter species distinguishes from these specimens by having the venter of mesosoma coriaceous, (consistently coriaceous-rugose in the other specimens), the fourth metasomal median tergite sculptured on basal half (always smooth in the other specimens), and the length between ocelli evidently longer. We also found considerable variation in some diagnostic features in the above specimens, suggesting there is more than one undescribed species involved, though we need to confirm their boundaries before describing any of them.

## Supplementary Material

XML Treatment for Notiospathius atra

XML Treatment for Notiospathius caudatus

XML Treatment for Notiospathius diversus

XML Treatment for Notiospathius johnlennoni

XML Treatment for Notiospathius novateutoniae

XML Treatment for Notiospathius sulcatus

XML Treatment for Notiospathius xanthofasciatus
